# Machine learning techniques for non-destructive estimation of plum fruit weight

**DOI:** 10.1038/s41598-024-85051-2

**Published:** 2025-01-04

**Authors:** Atefeh Sabouri, Adel Bakhshipour, Mehrnaz Poorsalehi, Abouzar Abouzari

**Affiliations:** 1 Department of Agronomy and Plant Breeding, Faculty of Agricultural Sciences, University of Guilan, Rasht, Iran; 2 Department of Biosystems Engineering, Faculty of Agricultural Sciences, University of Guilan, Rasht, Iran; 3 Crop and Horticultural Science Research Department, Mazandaran Agricultural Resources Research and Education Center, Agricultural Research, Education and Extension Organization (AREEO), Tajrish, Iran

**Keywords:** Artificial intelligence, Image processing, Fruit dimensions, Regression models, Computational biology and bioinformatics, Image processing, Plant sciences

## Abstract

Plum fruit fresh weight (FW) estimation is crucial for various agricultural practices, including yield prediction, quality control, and market pricing. Traditional methods for estimating fruit weight are often destructive, time-consuming, and labor-intensive. In this study, we addressed the problem of predicting plum FW using artificial intelligence (AI) methods based on fruit dimensions. We aimed to evaluate various machine learning (ML) techniques for this purpose. Images of fruit samples were captured using a smartphone camera, processed to extract binary images, and used to calculate dimensions. We tested several ML methods, including Support Vector Regression (SVR), Multivariate Linear Regression (MLR), Multi-Layer Perceptron (MLP), and Decision Tree (DT). The SVR model with a Pearson-VII kernel (PUK) function and penalty value (c) of 0.1 was the most accurate, achieving an R^2^ of 0.9369 and root mean squared error (RMSE) of 0.4850 (gr) during training, and 0.9267 and 0.4863 (gr) during testing. This method is important for researchers and practitioners seeking efficient, quick, and non-destructive ways to estimate fruit weight. Future research can build on these findings by applying the model to other fruit types and conditions.

## Introduction

Plums belong to *Prunus genus* and include a wide range of variations in shape and fruit size, color, aroma, and flavor. Although this plurality within “plums”, has only two types, the hexaploid (2n = 6x = 48) European plums (*Prunus domestica* L.) and the diploid (2n = 2x = 16) Japanese plums (*Prunus salicina* Lindl.), both are widely cultivated across the world^1^. Among the various kinds of plums, the Greengage (*Prunus domestica subsp. italica* var. *claudiana*.) hold a special place due to its unique characteristics and historical importance. Greengage originated in the Middle East^2^. Greengage is renowned for its unique flavor and health benefits, making it valuable in alcoholic beverages and cosmetic products. Recent studies highlight its nutritional value, bioactive compounds, and versatile applications. Rich in phenols and flavonoids, Greengage boasts significant antioxidant properties^3^. Key bioactive compounds such as linalool, terpineol, and chlorogenic acid exhibit strong antibacterial and antioxidant capacities^3^.

Due to the coincidence of the center of diversity of this species and the origin of the first human civilizations, it was one of the first fruits to be regarded by humans^4^.

The natural intraspecific crosses and long history can be one of the reasons for the creation of more than 2,000 varieties of this genus^5–7^. Plums are beneficial for human health due to their high content of antioxidant and phenolic compounds, dietary fiber, isatin, lutein, cryptoxanthin, zeaxanthin sorbitol, potassium, fluoride, iron, as well as vitamins A, C, and B-complex groups like vitamin B-6, pantothenic acid, and niacin. These compounds make plums effective in the prevention and treatment of various diseases^5^, including diabetes, cardiovascular diseases, obesity, and certain types of cancer^8–10^.

There is limited information available on Iran’s native wild fruit trees, and only a few cases have been reported in this area^7^. Cherry plum (*Prunus divaricata* Ledeb.) is a wild plum species that is diploid and can grow as a thornless deciduous shrub or tree up to 10 m tall. It is self-incompatible and produces edible fruits. This species is native to the Balkan Peninsula, Central Europe, Caucasus, and Central Asia, including northern Iran^11^. Regarding the conditions for planting and growing cherry plum in Iran, loam or sandy loam soils are preferred for optimal growth. Ideal planting occurs in late autumn or early winter, with a maximum planting distance of 5 to 6 m between trees. Base fertilizers should be applied, with additional topdressing throughout the year^12^.

*Prunus divaricata* Ledeb., along with other non-wood forest products and forest fruits, plays a significant role in the economy of rural households living in the forests of northern Iran, both directly and indirectly. It is commonly used as a spring fruit and as a rootstock to enhance resistance to root-knot nematodes in peach and plum nurseries. This makes it a suitable candidate for further research and domestication^6^.

Reproductive growth in fruit trees is primarily determined by fruit growth, and understanding this process is crucial for improving fruit production. Various horticultural treatments, such as regulated deficit irrigation, fruit thinning, and pruning, can be applied based on knowledge of plant growth patterns and predictions of fruit size parameters^13^. Fruit growth involves irreversible increases in volume and weight, which are influenced by both cell division and cell elongation. Fruit weight, volume, and diameter are important criteria for assessing fruit size^14^.

Monitoring these parameters can help determine optimal harvest time, estimate yield, identify varieties, and detect physiological disorders like fruit cracking. Fruit size used as an index to identification of the varieties^15^. Mathematical models have been developed to predict plant component growth, providing reliable criteria for plant nutrition, product quality, and yield rates. Measuring fruit weight can be done using both destructive and non-destructive methods. However, destructive methods that require harvesting leaves and fruits can be costly, and time-consuming, and limit the ability to study growth trends throughout the season, potentially reducing assessment accuracy^14,16^.

Artificial intelligence (AI) refers to the ability of a digital computer or a computer-aided robot to execute tasks usually performed by intelligent beings^17^. Image processing, a branch of AI, involves tasks such as preprocessing and segmentation image data, feature extraction, image classification, and object detection^18^. Tripathi and Maktedar^19^ reviewed several applications of computer vision and image processing in horticulture products.

Machine learning (ML), a trending subdomain of AI, is a rapidly growing discipline that enables computers to learn (be trained) from existing data and experiences without the need for explicit programming, and utilize the acquired knowledge to make predictions^20,21^. ML tools are utilized to explore, extract, analyze, and derive valuable information from data gathered from the real world^22^Citing several recent valuable review articles, the ML algorithms have proven to be efficacious in a wide array of agricultural and horticultural domains^23–26^. Common classical ML algorithms used in agro-related fields include Regression, Decision Trees (DTs), Artificial Neural Networks (ANNs), and Support Vector Machines (SVMs)^27^.

Soft computing algorithms and image processing techniques have been successfully employed for smart, non-destructive leaf area measurement of plum fruits based on their length and width^28^. Basak, et al.^29^ utilized image processing and ML methods to non-destructively estimate the weight of strawberries using Support Vector Regression (SVR) and Linear Regression (LR) algorithms. Ying-kai, et al.^30^ effectively estimated the weight of dragon fruits based on their dimensions extracted through image processing technique and an ANN model with the R^2^ and RMSE values of 0.986 and RMSE of 13.091 (gr), respectively. Image processing and LR methods were effectively employed by Ting-ting et al.^31^ for predicting tomato weight.

Huynh, et al.^32^ proposed a vision-based method for estimating the volume and mass of sweet potatoes using a simple vision system with a single camera to capture top-view images. The method involves segmenting the background and virtually slicing the product into equal sections along its longitudinal axis. The volume is calculated by summing the volumes of these individual slices, and the weight is estimated based on the high correlation between weight and volume. The approach achieved high accuracy, with up to 96% accuracy (R^2^ = 0.98) for volume estimation and up to 95% accuracy (R^2^ = 0.96) for weight estimation. This simple and effective model can be utilized in designing and developing systems for sizing, weighing, and packaging fruits and vegetables.

Fitriyah^33^ analyzed the accuracy of various methods to estimate the volume and weight of symmetrical and non-symmetrical fruits using computer vision. The study involved taking images of tangerines (symmetrical) and strawberries (non-symmetrical), processing these images using segmentation in saturation color space to obtain binary images. Regression methods utilized features such as Diameter, Projection Area, and Perimeter extracted from the binary images. For symmetrical fruits, the Linear Regression based on Diameter achieved the highest accuracy for both volume and weight, with the lowest errors. For non-symmetrical fruits, the highest accuracy was obtained using Linear Regression based on Diameter and Linear Regression based on Area, both showing strong performance for volume and weight estimation of strawberries.

Koç and Kayra^34^ developed a non-destructive weight prediction model for spherical fruits and vegetables, such as watermelons, melons, apples, oranges, and tomatoes, using data obtained from their images. The images were segmented using the U-Net architecture, and various machine learning models, including Multi-Layer Perceptron (MLP), Random Forest (RF), DT, SVM, Linear, and Stochastic Gradient Descent (SGD) regression models, were employed for weight predictions. The RF and DT models were found to be the most effective, achieving high success rates of 0.9112 for watermelon, 0.9944 for apple, 0.9989 for tomato, and 0.9996 for orange.

However, despite the capabilities of machine learning algorithms, there has been a lack of effective use of this potential for the indirect and non-destructive estimation of plum fruit weight through their images and ML algorithms. Previous studies have primarily focused on a narrow range of fruit varieties and may lack generalizability across different fruit types and conditions. Additionally, many existing models, while effective, may not provide comprehensive evaluations of model performance. Our study aims to address these gaps by including plums and greengages, developing robust models that accurately estimate fruit weight across various plum varieties, and using multiple machine learning algorithms and comprehensive evaluation metrics to ensure robust model performance.

This study aims to address the following research questions: What are the most effective mathematical models for estimating plum fruit weight? How can machine learning algorithms be utilized to create a comprehensive model for accurately estimating the weight of plums and greengages based on their dimensional characteristics? Therefore, we conducted this study with the following objectives:

1. To determine various mathematical models for estimating fruit weight and identify the most effective approach
2. To create a comprehensive model for accurately estimating fruit weight in plum and greengage using ML algorithms.

## Materials and methods

### General research methodology

**Fig. 1:**
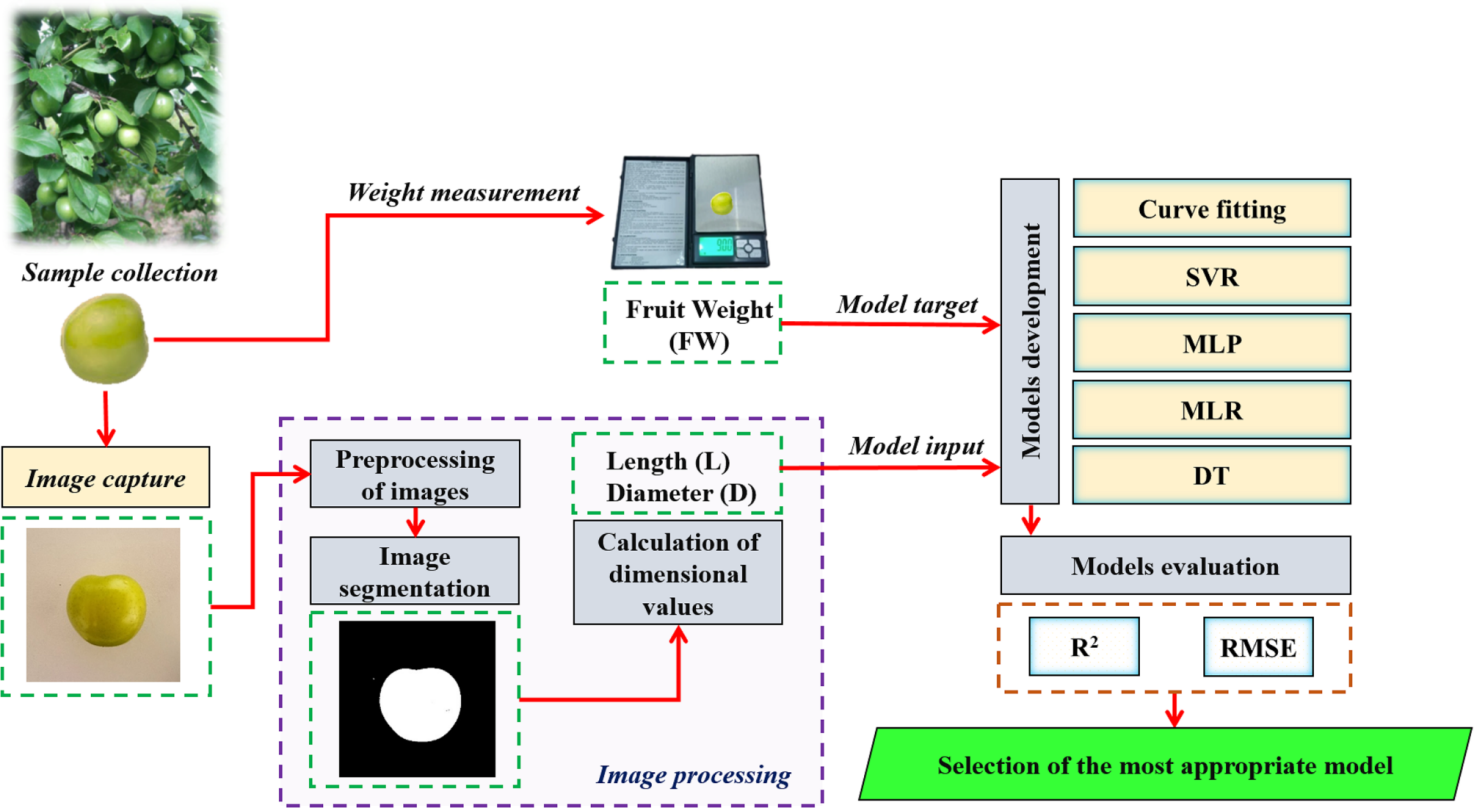
Diagram of the research methodology.

The methodology of this research is outlined in Fig. 1. The study integrates image processing techniques and machine learning algorithms to estimate the mass of plum fruits in a non-destructive manner. Data collection was conducted in 2019 and 2021, where plum samples were photographed using a smartphone under natural ambient lighting. Simultaneously, the mass of each sample was determined with a digital balance. The acquired images were analyzed, and by converting them to binary format, the two key dimensions—length and diameter—of the plum samples were extracted from the segmented images.

Several predictive models were constructed and assessed, including SVR, MLP, DT, Multiple Linear Regression (MLR), and a curve fitting method. These models were developed to determine the most accurate approach for fruit mass estimation. The length and diameter of the fruits served as input features, while the sample masses represented the target variable. The optimal models were selected based on achieving the highest R^2^ and the lowest RMSE, ensuring robust and reliable predictions. These methodology steps are detailed in the following sections.

### Sampling and fruits data

**Fig. 2:**
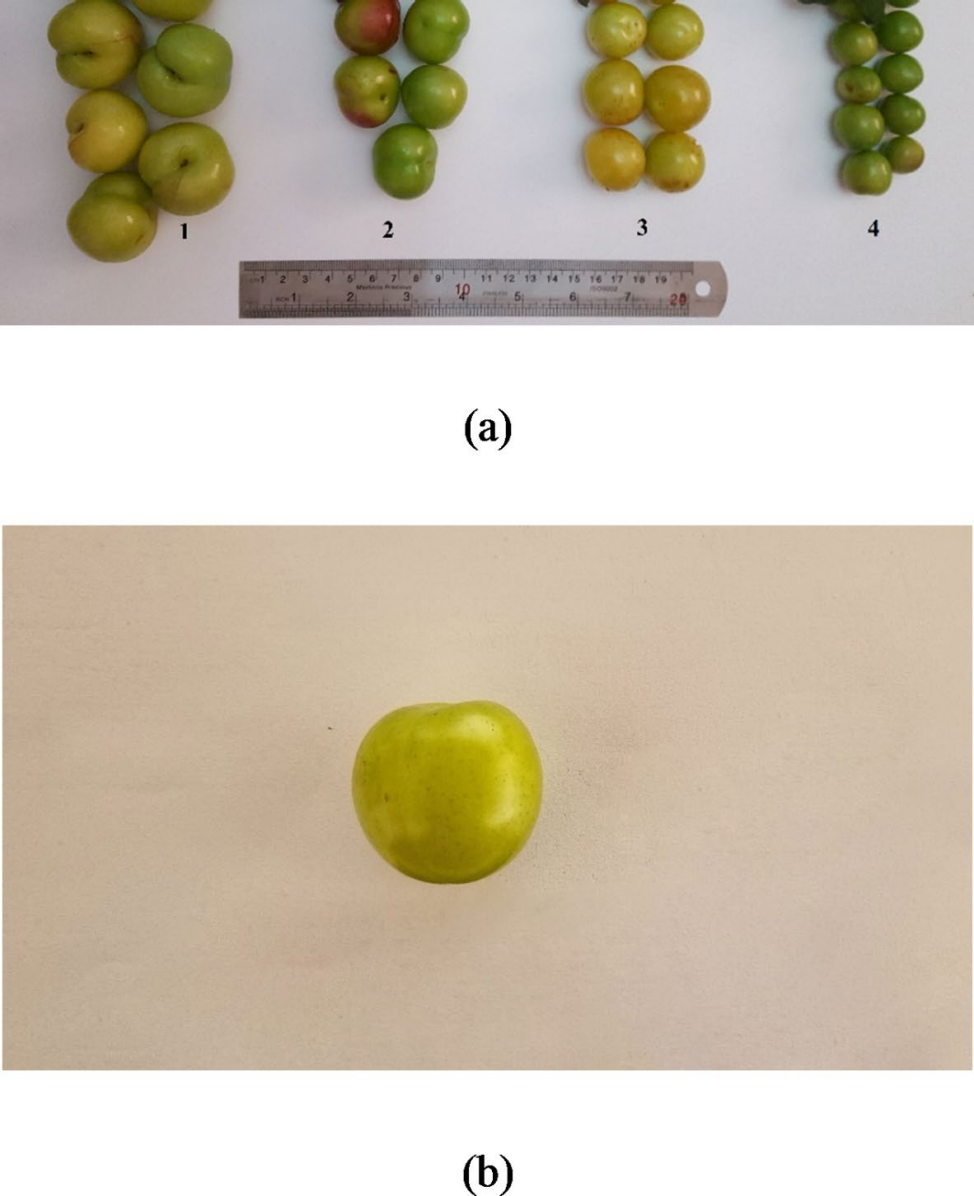
(**a**) The fruits of three greengage [*Prunus domestica* (subsp. *italica* var. *claudiana*.)] genotypes (No. 1–3) with local names Gavali, Ghandi and Shahryari as long as a myrobalan plum (*Prunus ceracifera*) with local name of Jangali (No. 4), and (**b**) a sample image of a single fruit used for image processing. (photos were taken by authors 2019–2021)

To develop and validate a model for estimating fruit weight, we used three greengage genotypes (*Prunus domestica* subsp. italica var. claudiana) named Gavali, Ghandi, and Shahryari, as well as a myrobalan plum (*Prunus ceracifera*) called Jangali (Fig. 2a). These genotypes are commonly found in the northern region of Iran. The trees ranged in age from 15 to 26 years. After harvesting, we placed the fruit samples in a cooler and immediately transported them to the laboratory for data collection.

The first experiment took place during the 2019 growing season. We collected 1,028 undamaged Gavali greengage fruits from the research orchard located at the Faculty of Agricultural Sciences, University of Guilan in Rasht, Iran (37°16’N, 51°3’E).

**Fig. 3:**
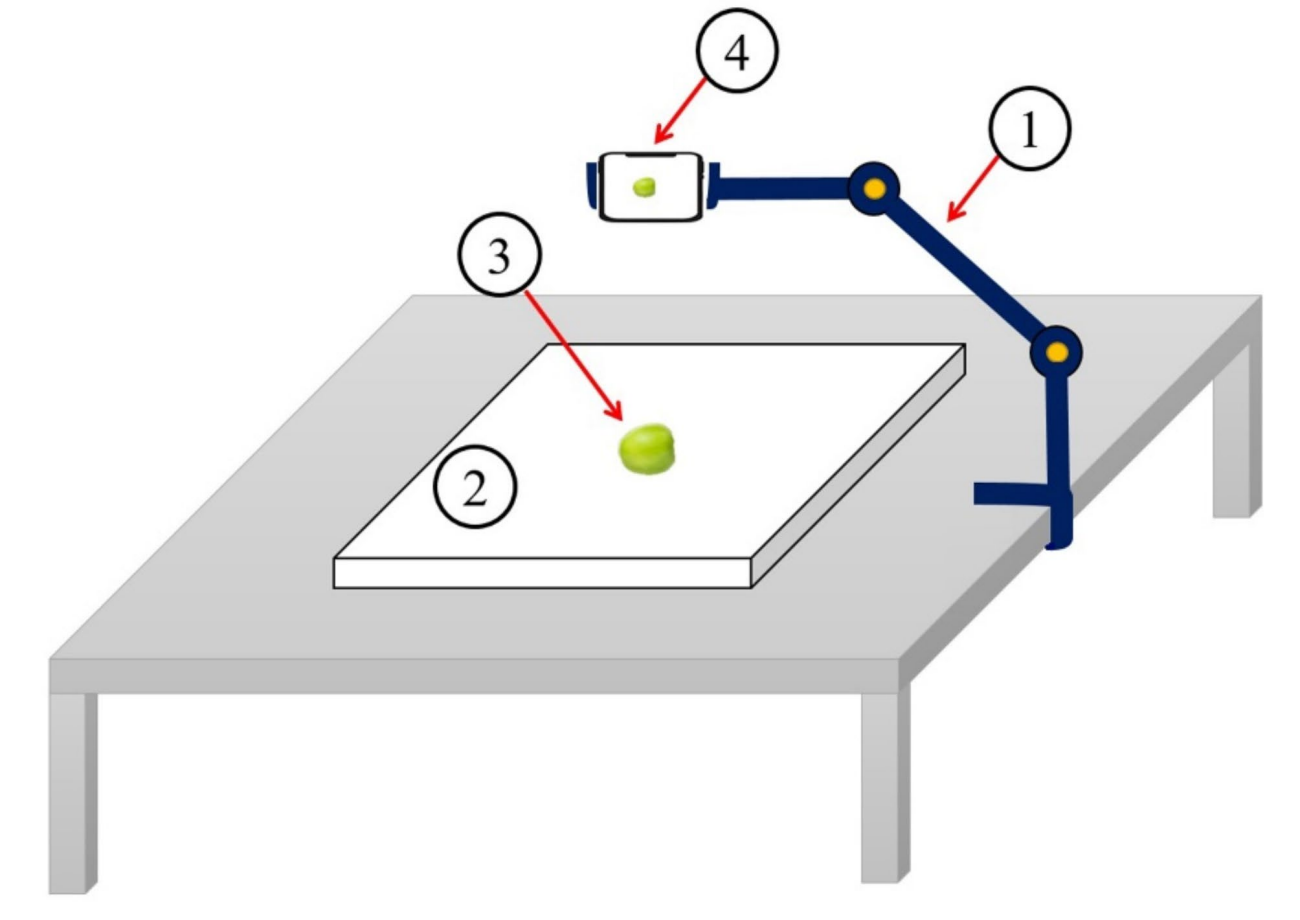
A schematic view of the imaging setup, including a stand frame (1), a stage (2), sample plum (3), and a smartphone (4).

The fruit fresh weight (FW) measurements were determined by using a technical balance (Notebook Series KIA Digital Scale, Accuracy 0.01 gr, China). Concurrently, the images of the fruit samples were captured using a smartphone camera to determine their dimensions using image processing techniques. Each fruit was placed individually on a matte white paper, and the phone lens was positioned 10 cm directly (vertically) above the sample (Fig. 3). All fruit samples were positioned in a consistent orientation on the stage to minimize potential errors in dimension extraction.

The utilized smartphone was Samsung Galaxy S21 FE that has a main camera with a 12MP resolution (camera name: SM-G990E, sensor type: CMOS, sensor name: IMX555 camera sensor) and the images were captured at size of 3200 × 1800 pixels and ISO of 300. These manual camera settings were used to eliminate or minimize the effect of phones’ built-in algorithms on the images. The images of the fruit samples were captured during morning (9–12 AM). No artificial illumination system used, and care was taken to avoid direct sunlight or shadows on the samples to ensure uniform lighting conditions (Fig. 2b). The images were then transferred to a computer for processing and feature extraction.

All methods were performed in accordance with the relevant institutional, national, and international guidelines and legislation. All international, national and institutional guidelines have been taken into account in various stages of experiments.

### Image processing and dimension extraction

**Fig. 4:**
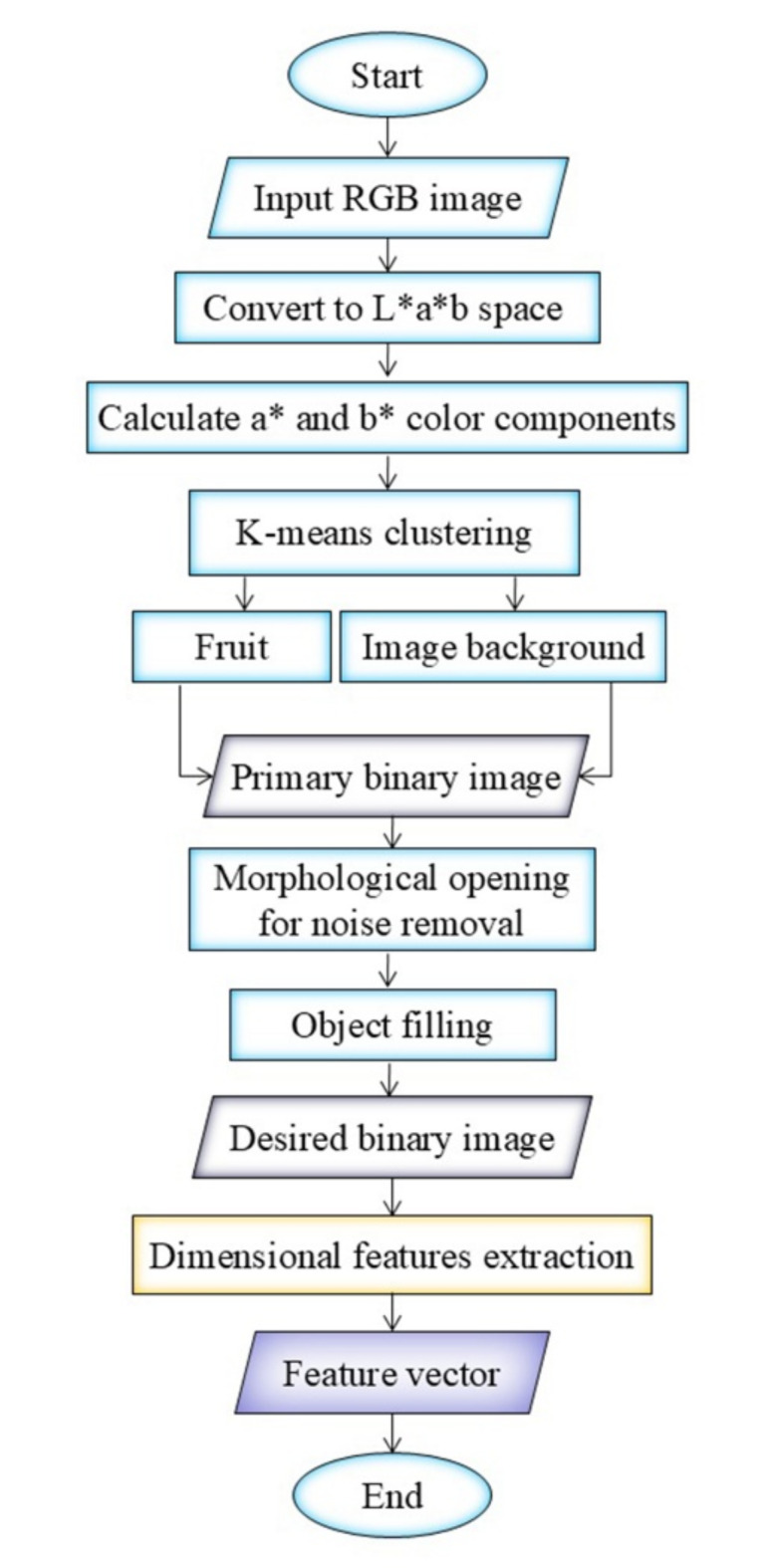
Flowchart of image processing steps for extracting dimensional data from plum images.

**Fig. 5:**
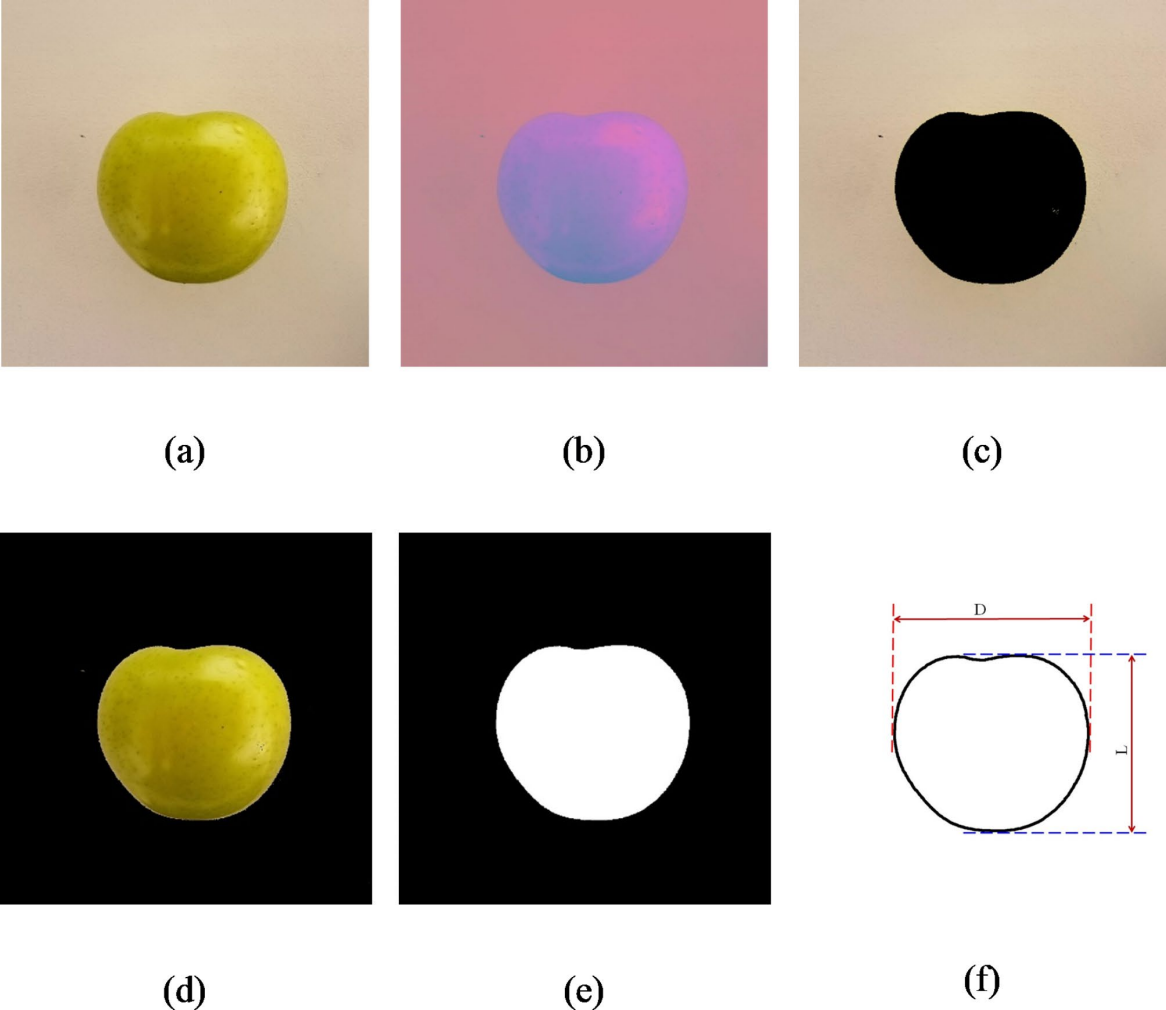
Image processing steps, (**a**) primary RGB image of plum fruit, (**b**) L*a*b image of plum fruit, (**c**) cluster showing the image background, (**d**) cluster showing the plum fruit, (**e**) binary image of plum fruit, and (**f**) Diagram illustrating the definition of extracted dimensions. Note: to better visualize the algorithm results, the images in Fig. 5 are displayed in a 1200 × 1200 pixels cut block.

We processed the plum fruit images using the image processing toolbox in MATLAB software (The MathWorks, USA, version R2021a). A flowchart of image processing steps is presented in Fig. 4, while Fig. 5 shows the results of different stages of image processing. The color of plum fruits can vary from green to yellow to red, therefore, applying simple thresholding on the color components was ineffective for segmenting images of fruits with different colors. To address this issue and develop a more comprehensive algorithm, we implemented a new segmentation algorithm based on color-based K-means clustering to separate the fruit from the image background (using 2 clusters). K-means clustering is an unsupervised algorithm that classifies a set of N observations into k clusters by iteratively performing the following steps: (1) choosing k cluster centers, and (2) assigning observations to the closest cluster by minimizing the distance of each observation from the cluster center^35^.

Since the images were captured under natural ambient light conditions, we transformed the RGB images (Fig. 5a) into the L*a*b color space (Fig. 5b) to minimize the impact of varying light radiation on the image segmentation results. The L component, which denotes the lightness of the image, was not used in the clustering process. Instead, the a* and b* chromatic components were utilized as input variables for the k-means clustering algorithm. Clusters related to the plum fruit and the image background are depicted in Fig. 5c and d, respectively. The binary image of plum was generated from the plum cluster image. To eliminate potential noise, morphological opening (dilation followed by erosion) was applied using a disk-shaped structural element with a radius of 5 pixels through MATLAB’s “*imopen*” function. Subsequently, any holes within the Region of Interest (ROI) were filled using MATLAB’s “*imfill*” function to obtain the final binary image of the plum fruit (Fig. 5e). The main dimensions of fruits (as defined in the Fig. 5f) were extracted from the binary image. The terms L and D in Fig. 5f refer to the maximum length and maximum diameter in the equatorial plane, respectively, extracted from images of the fruits. Eventually, the image of a 10 × 10 mm black square under equal imaging conditions was used to calculate the conversion factor from pixels to millimeters and used for fruit weight estimation.

### Calibration of a mathematical models

To find an appropriate mathematical model to estimate the weights of plum fruits based on their image-based length and diameter values, we developed and evaluated five different combinations of fruit length (L), and diameter (D) as independent variables: (1) FW = a + b×(L×D), (2) FW = a + b× L^3^, (3) FW = a + b×D^3^, (4) FW = a + b×(L× D^2^), and (5) FW = a + b×(L^2^× D), where a and b are constants calculated for each formulation. We performed mathematical modelings using Curve Fitting Toolbox in MATLAB programming software. The curve fitting algorithm determines the constants and coefficients (a and b in this study) of these mathematical functions to achieve the best fit for the data points. The fitting process in the MATLAB curve fitting toolbox utilizes the least squares method, tuning the parameters to minimize the root mean square error (RMSE).

### Machine learning algorithms

In addition to mathematical models, we developed different structures of four well-known machine learning methods for regression modeling in FW prediction based on L and D: Support Vector Regression (SVR), Multiple Linear Regression (MLR), Multilayer Perceptron (MLP), and Decision Trees (DT). The L and D values of the plum fruits were input into the ML models as predictors, while the FW measures served as the response variables.

#### SVR model

SVR is a modified version of Support Vector Machines (SVM) for regression algorithms. Like SVMs, the hyperplane generation is performed in SVR, where the best-fitted line is the hyperplane with the maximum number of data points^36^. Two important factors that impact the SVR performance are the kernel type and penalty value (c)^37^. A kernel function is a computational tool that transforms input data into a higher-dimensional feature space, enabling complex patterns to be separable by a hyperplane^38^. The c parameter represents the tolerance to error, influencing the model’s fitting endpoint^39^. In this study, we used SVR models with three kernel types (Radial Basis Function (RBF), Pearson VII kernel (PUK), and 2D polynomial) and three c values (0.1, 1, and 10), which are commonly used in SVM models^40,41^.

#### MLP model

MLP is an artificial neural network (ANN) with a feed-forward learning approach and error back-propagation training algorithm, consisting of an input layer, one or more hidden layers, and an output layer^42^. Feed-forward indicates that information flows solely in one direction, from the input nodes to the hidden nodes, and then to the output nodes^43^. The term back-propagation refers to the process where, if the network fails to achieve the expected performance during training, the error is propagated back to retune the network parameters^44^. In this study, we used a simple MLP with one hidden layer and different number of neurons in the hidden layer (1 to 20 neurons), and tangent sigmoid transfer function, which is commonly used in regression MLPs.

#### MLR model

MLR is a popular ML technique aimed at statistically find the best linear equation between a dependent response and two or more independent variables^45^. MLR creates different linear regression equations by combining predictor variables to model a dependent variable and the optimal equation is selected based on the highest correlation coefficient and the smallest sum of squared residuals^46^. In this study, the optimal values of intercept (β0) and slope coefficients (β1 and β2) in the general form of FW = β0 + β1L + β2D were investigated to generate the most accurate MLR model.

#### DT model

DTs can also be used to predict continuous variables in regression problems. In these cases, the DTs are also called regression trees^47^. In this structure, the tree leaves predict the continuous response variable based on the linear regression algorithm^48^. In this study, we evaluated three different DT algorithms: M5P, Random Forest, and Reduced Error Pruning (REP) for FW prediction based on fruit L and D values. Descriptions about these Models are available in literature^49,50^.

### Evaluation and validation of calibration models

To develop the models, we randomly divided the datasets into two categories: training (70%) and testing (30%). For example, from a collection of 1,028 samples in 2019, 720 samples were randomly selected for model training, while the remaining 308 samples were used for model testing. The number of randomly separated samples from Gavali, Ghandi, Shahryari, and Jangali plums in 2021 for model training were 363, 356, 371, and 340, respectively.

The training process of the machine learning models was done based on 10-fold validation strategy. To choose the best structure of each model and to compare between models, we compared the estimated FW values with the corresponding actual FW values. This was done by calculating two statistical criteria: Root Mean Squared Error (RMSE) and the coefficient of determination (R^2^) between measured and estimated FW data. These two statistics are calculated using Eqs. (1) and (2)^51^.

1 $$\:\text{R}\text{M}\text{S}\text{E}={\left[\frac{1}{\text{n}}\sum\:_{\text{i}=1}^{\text{n}}{({\text{F}\text{W}}_{mea,i}-{\text{F}\text{W}}_{est,i})}^{2}\right]}^{0.5}$$

2 $$\:{\text{R}}^{2}=1-\left[\frac{\sum\:_{\text{i}=1}^{\text{n}}{({\text{F}\text{W}}_{mea,i}-{\text{F}\text{W}}_{est,i})}^{2}}{\sum\:_{\text{i}=1}^{\text{n}}{({\text{F}\text{W}}_{mea,i}-\stackrel{-}{{\text{F}\text{W}}_{mea}}\:)}^{2}}\right]$$

where $\:{\text{F}\text{W}}_{est,i}$ and $\:{\text{F}\text{W}}_{mea,i}$ are the *i*th estimated and measured FW values, $\:\stackrel{-}{{\text{F}\text{W}}_{mea}}$ stands for the average of the measured FW values, and n is the total number of samples. The lower the RMSE and the higher the R^2^ value (closer to 1), the more accurate predictions is achieved by the model. Additionally, the regression lines of actual versus predicted FW data should not significantly deviate from the 1:1 line, which has a slope of 1 and an intercept of 0 ^52^.

Furthermore, to evaluate the generality of the developed model, we conducted a second experiment in 2021 using four genotypes. One genotype was the same as the one used for developing the model (greengage Gavali) and three additional genotypes included Ghandi and Shahryari genotypes from greengage (*prunus domestica*) and Jangali genotype from plum myrobalan (*prunus ceracifera*). In this regard, 518, 530, 508, and 486 fruits were sampled for Gavali, Shahryari, Ghandi and Jangali genotypes during June 2021. We determined the maximum diameter, equatorial diameter, and actual fruit weight of the selected samples according to the preliminary method. The RMSE and R^2^ were also calculated to assess the goodness-of-fit and validity of the developed model on the new datasets.

## Results and discussion

### General statistical analysis

Table 1 presents the descriptive statistics for fruit L, D, and FW of four plum genotypes based on data collected in 2019 and 2021. The data indicates variability in the fruit length, diameter, and weight among the different plum genotypes. The 2019 dataset (Gavali) shows a relatively higher mean fruit weight compared to the 2021 Gavali and other genotypes. Among the 2021 genotypes, Gavali has the highest mean fruit length and diameter, while Jangali has the smallest dimensions and weight. The standard error values suggest that there is some variability within each genotype.

**Table 1:** Descriptive statistics of fruit length (L), diameter (D) and fresh weight (FW) of plum datasets in 2019 and 2021 (SE: Standard Error of Mean).

Dataset name	dataset size	Fruit length (mm)			Fruit diameter (mm)			Fruit weight (gr)		
		Mean ± SE	Max	Min	Mean ± SE	Max	Min	Mean ± SE	Max	Min
2019 dataset	1028	25.74 ± 1.64	32.22	21.13	24.50 ± 1.84	30.55	19.17	9.74 ± 1.89	16.29	5.21
2021 Gavali	518	26.11 ± 1.96	30.88	20.44	25.73 ± 2.01	30.80	18.58	9.26 ± 2.05	14.77	3.72
2021 Ghandi	508	22.44 ± 2.06	26.97	15.92	21.95 ± 2.11	27.07	16.03	6.44 ± 1.76	11.80	2.34
2021 Shahryari	530	23.58 ± 2.31	29.22	16.74	21.78 ± 2.55	28.33	13.29	7.32 ± 2.34	14.38	1.60
2021 Jangali	486	15.12 ± 1.19	19.00	11.15	13.52 ± 1.19	16.66	8.22	1.44 ± 0.36	2.85	0.38

**Fig. 6:**
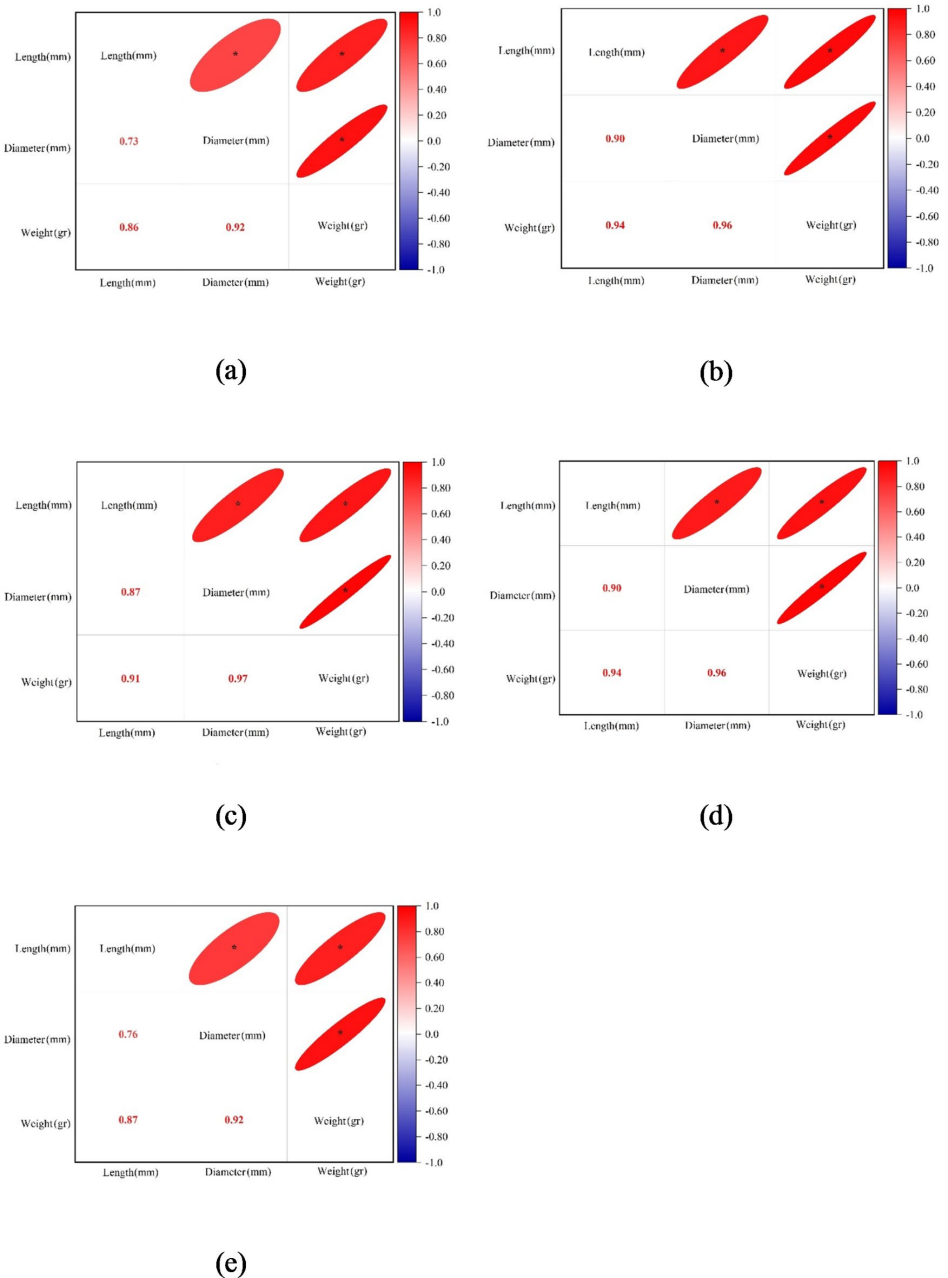
Correlation heat maps between plum fruit length, diameter, and weight values; (**a**) 2019 dataset (**b**) 2021 Gavali dataset, (**c**) 2021 Ghandi dataset, (**d**) 2021 Shahryari dataset, and (**e**) 2021 Jangali dataset (* stands for significance at 0.01 level).

The results of the Pearson correlation test in Fig. 6 show that there are significant (*P* < .01) correlations between the dimensional measures of plum fruits and their weight. Although the high intercorrelation between the length and diameter values ​​was expected, the correlation of each of the length and diameter values ​​with fruit weight was greater than the dimensional intercorrelation. This indicates that we can hope for the development of an accurate model for estimating the weight of plum fruits based on their dimensions.

**Fig. 7:**
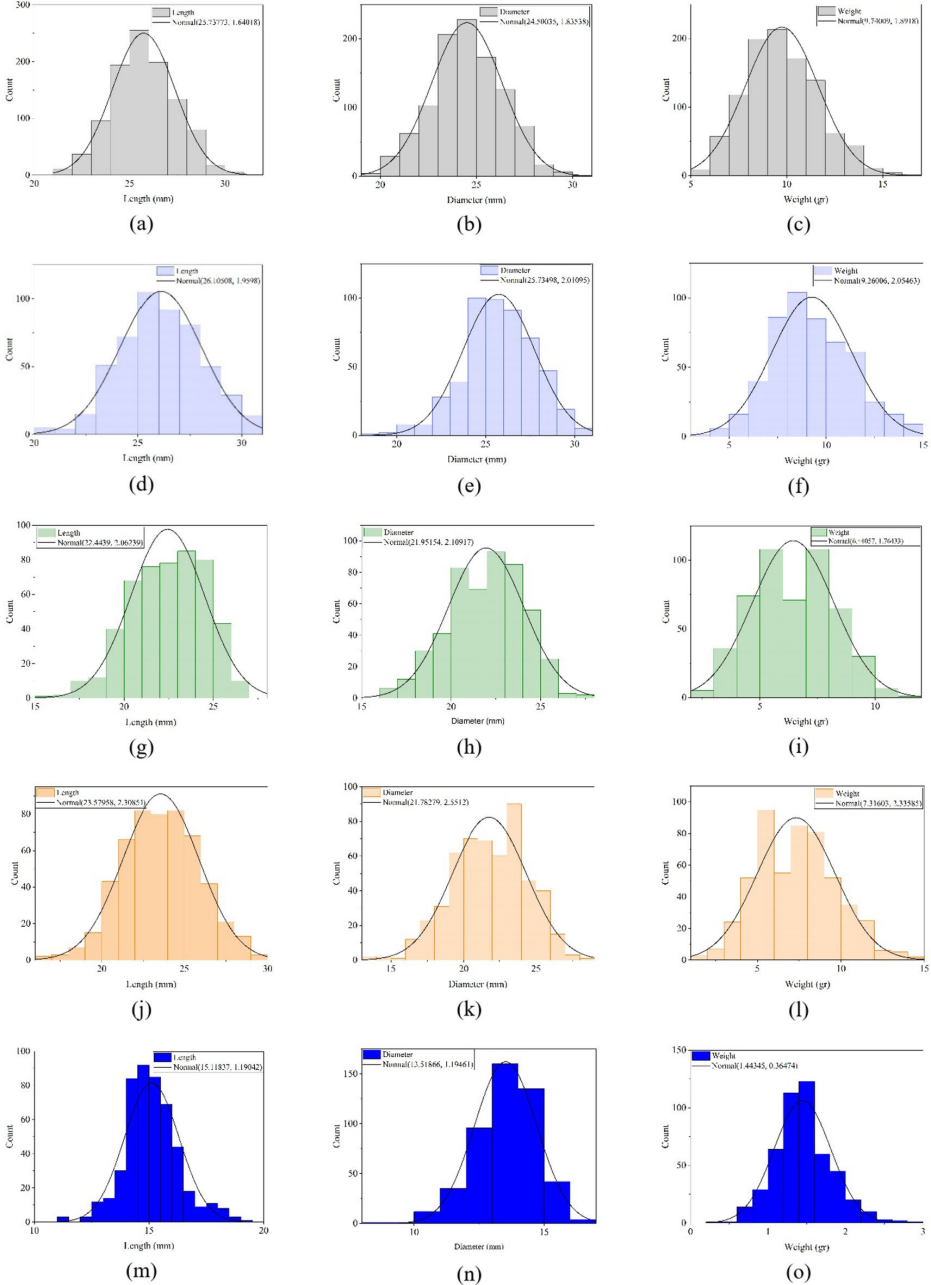
Statistical distribution plots of fruit length (L), diameter (D), and fresh weight (FW) for different plum types: (**a**–**c**) 2019 data, (**d**–**f**) 2021 data for Gavali plum, (**g**–**i**) 2021 data for Ghandi plum, (**j**–**l**) 2021 data for Shahryari plum, and (**m**–**o**) 2021 data for Jangali plum.

Histograms representing the distributions of fruit L, D, and FW values (Fig. 7) provide a clear visualization of the data’s spread and central tendencies. The plots indicate that the dimensional and weight measurements of the fruits generally exhibit a bell-shaped distribution, consistent with the characteristics of a normal distribution. These distributions feature a central peak with relatively symmetrical data spread on either side, with very negligible skewness values in some cases, reflecting the natural variation within the sampled fruits. Such insights help characterize the population and validate the representativeness of the dataset for subsequent analyses.

### Model fitting to 2019 dataset

Table 2 displays various mathematical equations used to estimate the fruit FW of the Gavali plum (*Prunus domestica*) in 2019. The most effective model was determined to be the product of both fruit length (L) and diameter (D) with the formula of FW = 0.0225(L×D)-4.4940, as it yielded the lowest RMSE value (0.4947 gr) and the highest R^2^ (0.9343) in the calibration stage. According to Table 2, the evaluation of this model on the separate test dataset resulted in RMSE and R^2^ values of 0.5046 gr and 0.9214, respectively. These values ​​confirm the reliable performance of the developed mathematical model in estimating the mass of plum samples. The second-rank best model was FW = 0.0006(L×D^2^) + 0.5766, with RMSE values of 0.5001 gr and 0.5071 gr in the training and test stages.

**Fig. 8:**
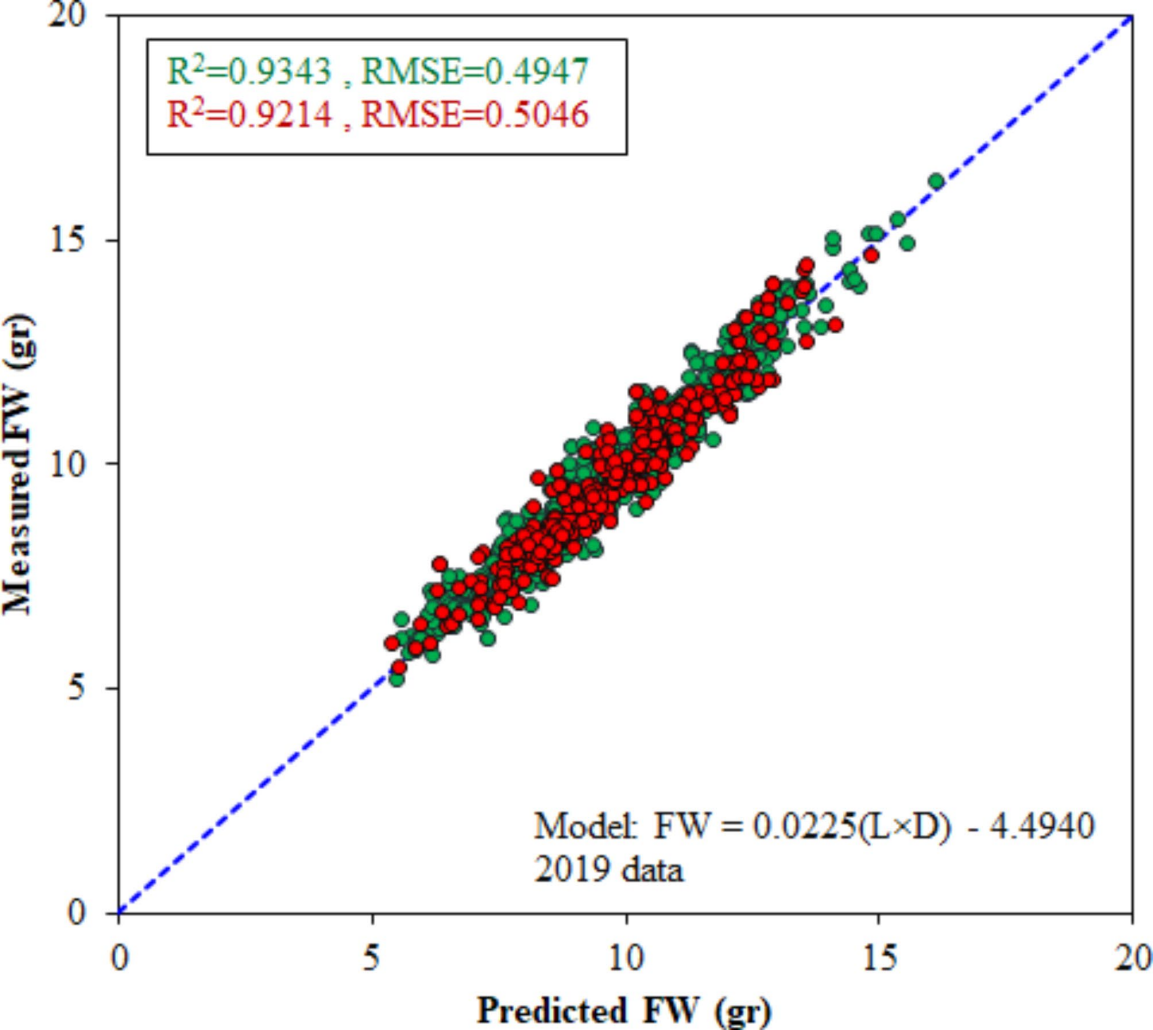
Scatter plot of FW = 0.0225(L×D)-4.4940 model for FW estimation in 2019 dataset during calibration (green dots) and test (red dots) stages.

On the other hand, the model that solely utilized fruit L resulted in the lowest R^2^ (0.7537) value and the highest RMSE (0.9593 gr), making it less reliable for estimating plum FW. Although the results of using alone the fruit diameter value performed slightly better than the length-based model, it still did not yield acceptable results. The measured vs. model-predicted values of the FW = 0.0225(L×D) − 4.4940 model results in the training and test stages on the 2019 dataset are depicted in Fig. 8. The close scatter of the data points around the 1:1 line demonstrates high agreement between the model-estimated and actual FW data.

**Table 2:** Results of the fitted models for predicting the weight of plum fruits (FW) from their length (L) and diameter (D) values in the 2019 dataset.

Model configuration	Model coefficients		RMSE (gr)		*R* ^2^	
	a	b	Train	Test	Train	Test
**FW = a + b(L×D)**	**-4.4940**	**0.0225**	**0.4947**	**0.5046**	**0.9343**	**0.9214**
FW = a + b(L^3^)	1.2440	0.0005	0.9593	0.9449	0.7537	0.7252
FW = a + b(D^3^)	1.8260	0.0005	0.7278	0.7099	0.8583	0.8446
FW = a + b(L×D^2^)	0.5766	0.0006	0.5001	0.5071	0.9331	0.9205
FW = a + b(D×L^2^)	0.2421	0.0006	0.5969	0.5907	0.9046	0.8922

### ML modeling of 2019 dataset

The specifications and performance measures of the optimal structures of SVR, MLR, MLP, and DT models for plum weight estimation in the 2019 dataset are available in Table 3. The RMSE and R^2^ statistics in Table 3 show that the optimal configurations of all investigated models had good results for estimating plum weight based on their length and width. It is also evident that the SVR model, with the lowest RMSE (0.4850 gr) and highest R^2^ (0.9369) in the training period, was the most accurate model for plum weight estimation in the 2019 dataset. The optimal c value and kernel type of this model were c = 0.1 and PUK kernel respectively. The RMSE and R^2^ criteria of the PUK-SVR model in the test stage were 0.4863 gr and 0.9267, respectively. Based on these criteria, the PUK-SVR model performed better than the fitted FW = 0.0225(L×D) − 4.4940 model.

**Fig. 9:**
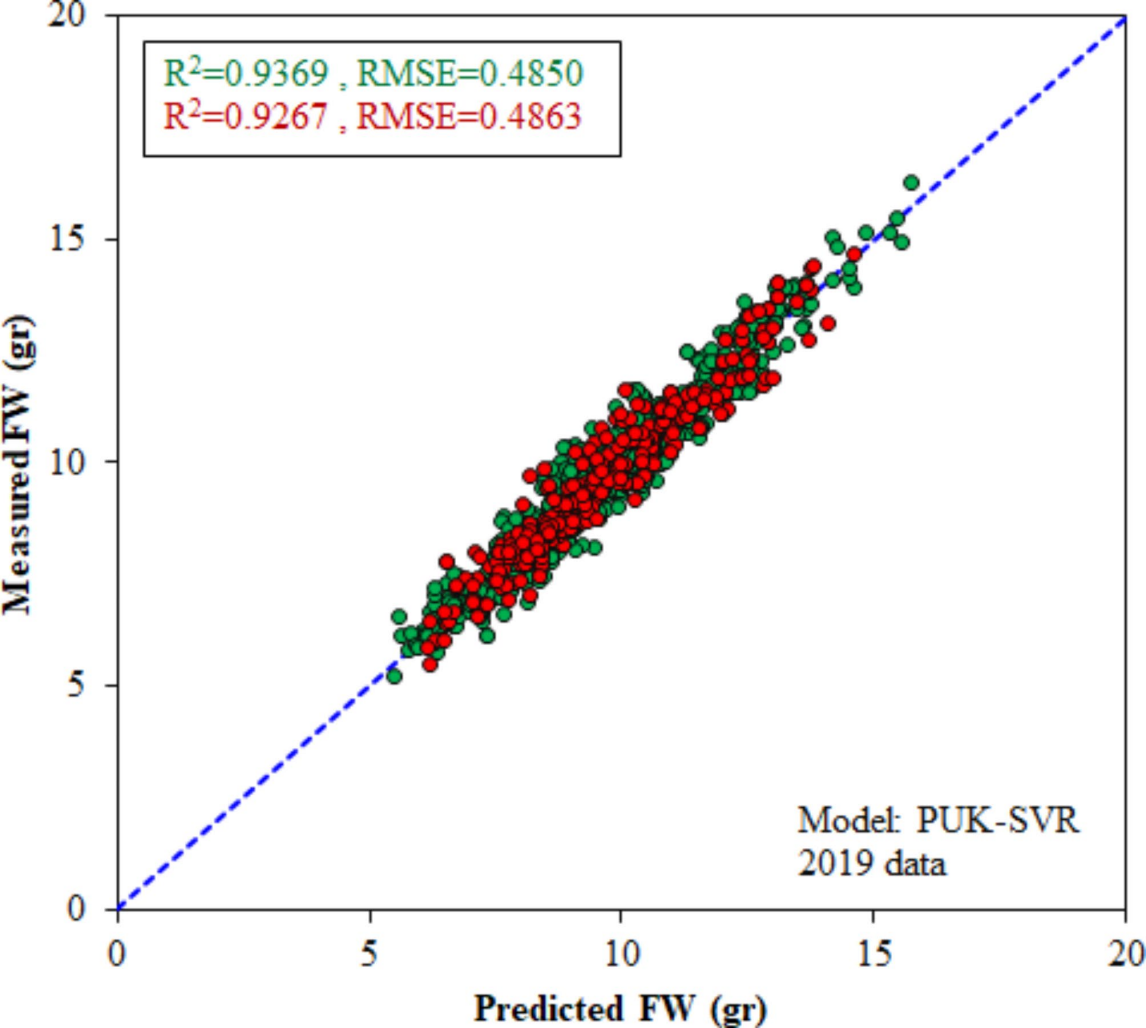
Scatter plot of the PUK-SVR model for FW estimation in 2019 dataset during calibration (green dots) and test (red dots) stages.

Another point that; although the MLP model was less accurate than other models in this research, this does not violate the high ability of ANNs in prediction problems. In this study, we employed almost the simplest structure of neural networks with only one hidden layer. Increasing the number of hidden layers or changing training parameters may improve the performance of ANNs. On the other hand, in cases where the number of input components is much higher than in this research, the capabilities of ANNs are revealed. Figure 9 shows the scattergram of the results obtained from the PUK-SVR model on the 2019 training and test datasets. The high density of data around the identity line demonstrates the high efficiency of the PUK-SVR model.

**Table 3:** The best SVR, MLR, MLP, and DT models for predicting the weight of plum fruits in the 2019 dataset.

Model	Specifications	RMSE (gr)		*R* ^2^	
		Train	Test	Train	Test
MLR	FW = 0.479 L + 0.644D -18.346	0.5033	0.5128	0.9320	0.9189
MLP	Topology: 2-3-1	0.5520	0.4817	0.9201	0.9278
DT	M5P Tree	0.5017	0.4883	0.9333	0.9264
**SVR**	**c = 0.1**,** PUK kernel**	**0.4850**	**0.4863**	**0.9369**	**0.9267**

### Model development for 2021 data

**Fig. 10:**
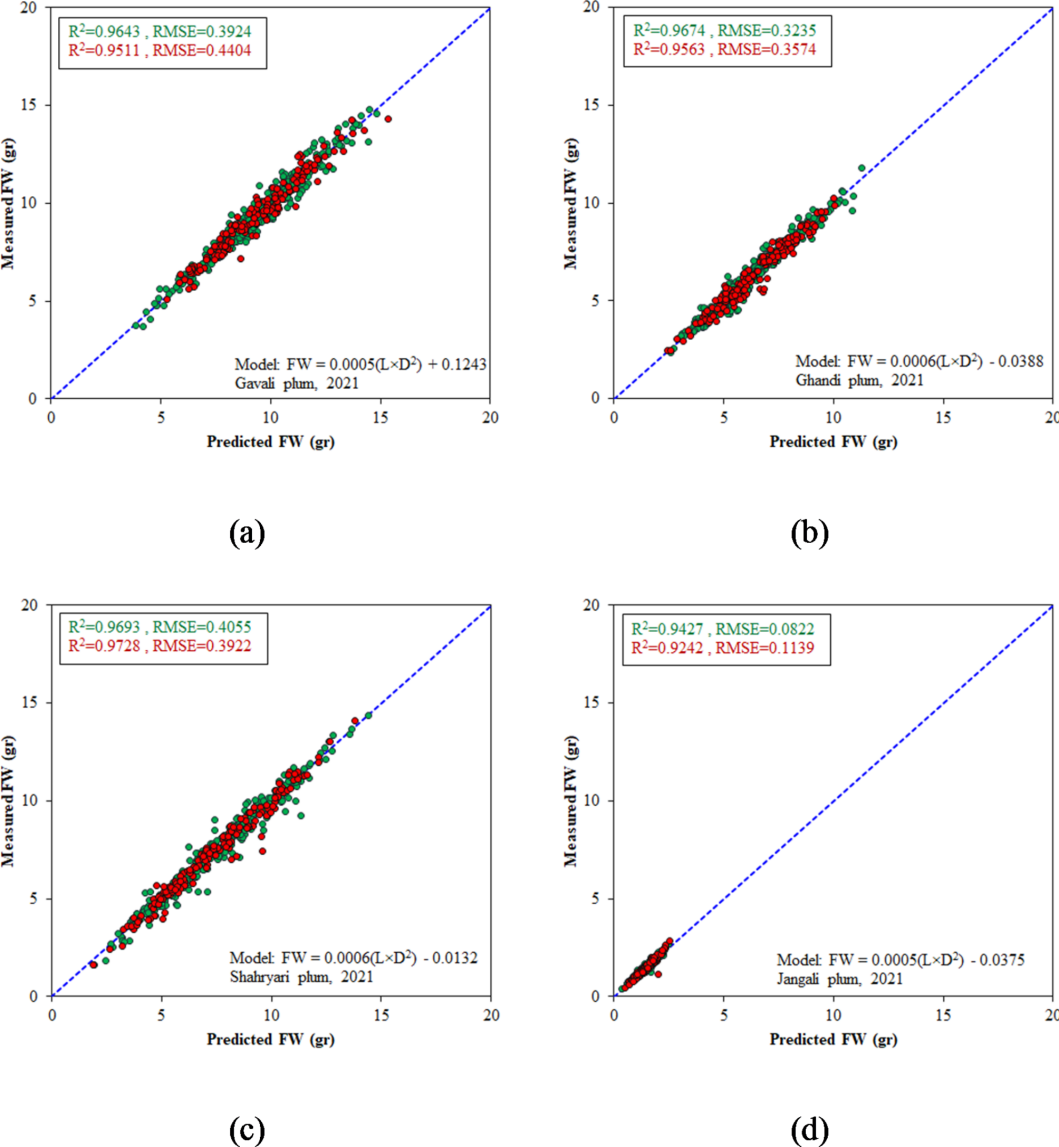
Scatter plots of the fitted models for FW estimation in 2021 dataset during calibration (green dots) and test (red dots) stages; (**a**) Gavali genotype, (**b**) Ghandi genotype, (**c**) Shahryari genotype, and (**d**) Jangali genotype.

**Fig. 11:**
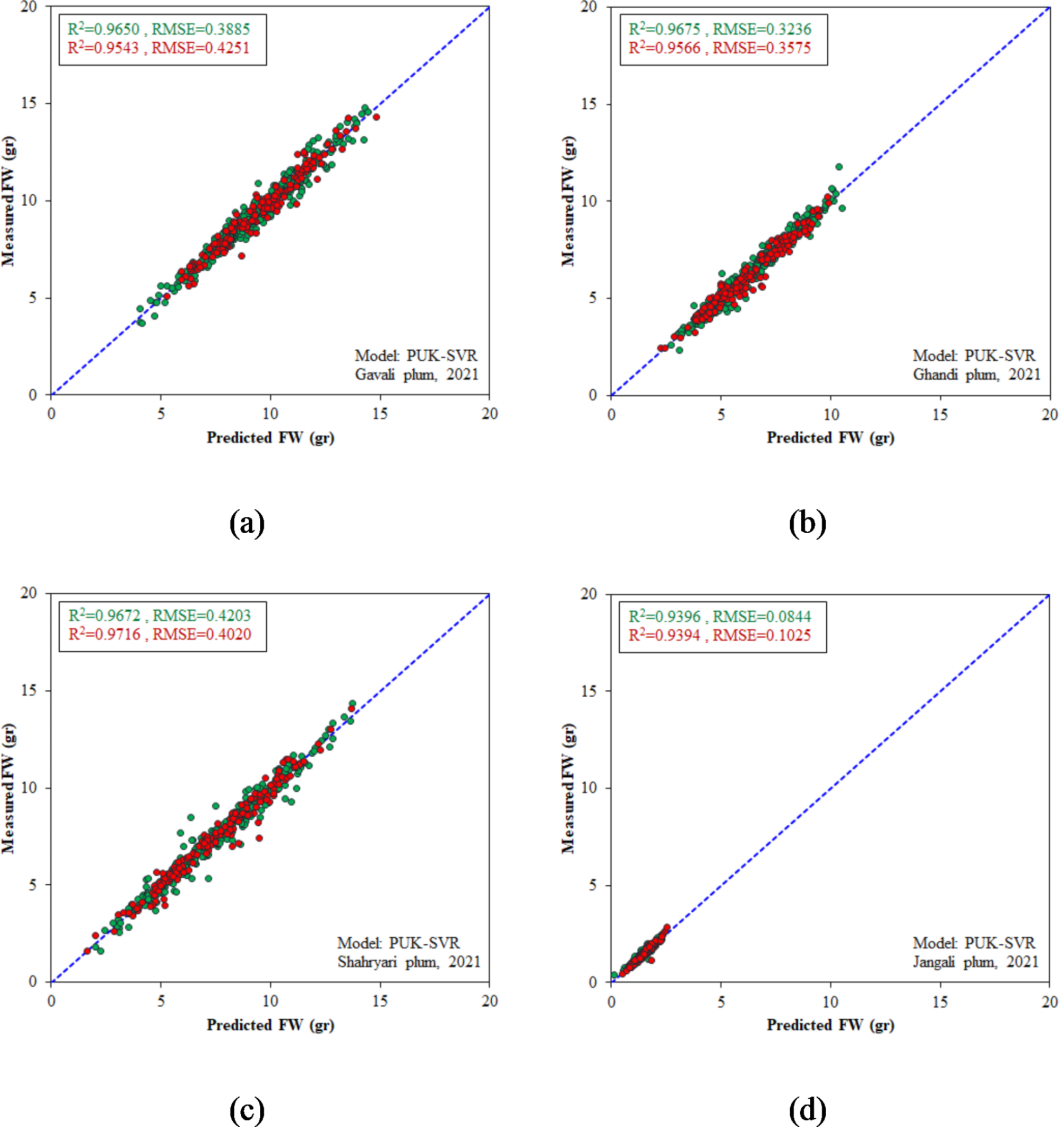
Scatter plots of the PUK-SVR models for FW estimation in 2021 dataset during calibration (green dots) and test (red dots) stages; (**a**) Gavali genotype, (**b**) Ghandi genotype, (**c**) Shahryari genotype, and (**d**) Jangali genotype.

As a secondary objective in this research, we also carried out the mathematical and intelligent modeling processes on the 2021 datasets. The characteristics and performance outcomes of the most successful structures of each model type are presented in Table 4. It is evident from Table 4 that all the optimal models were highly effective in estimating the weight of samples from different plum genotypes. In general, the fitted models and SVR models gave almost identical responses and were somewhat more accurate than the DT and MLR models. The scatter plots of the fitted models and SVR models for predicting the FW of different genotypes can be found in Figs. 10 and 11, respectively.

**Table 4:** The best models for predicting the weight of fruits from various plum genotypes in the 2021 datasets.

Genotype	Model	Specification	RMSE (gr)		R^2^	
			Train	Test	Train	Test
Gavali	Fitted model	FW = 0.0005(L×D^2^) + 0.1243	0.3924	0.4404	0.9643	0.9511
	MLR	FW = 0.452 L + 0.579D − 17.414	0.4436	0.4550	0.9543	0.9479
	MLP	Topology: 2-9-1	0.4472	0.5988	0.9547	0.9547
	DT	M5P Tree	0.4056	0.4270	0.9618	0.9539
	SVR	c = 1, PUK kernel	0.3885	0.4251	0.9650	0.9543
Ghandi	Fitted model	FW = 0.0006(L×D^2^) − 0.0388	0.3235	0.3574	0.9674	0.9563
	MLR	FW = 0.228 L + 0.622D − 12.323	0.3623	0.4112	0.9590	0.9432
	MLP	Topology: 2-3-1	0.3682	0.3871	0.9588	0.9551
	DT	M5P Tree	0.3278	0.3590	0.9665	0.9563
	SVR	c = 1, PUK kernel	0.3236	0.3575	0.9675	0.9566
Shahryari	Fitted model	FW = 0.0006(L×D^2^) − 0.0132	0.4055	0.3922	0.9693	0.9728
	MLR	FW = 0.358 L + 0.594D -14.062	0.4941	0.4843	0.9545	0.9584
	MLP	Topology: 2-4-1	0.4302	0.4126	0.9655	0.9732
	DT	M5P Tree	0.4211	0.4174	0.9669	0.9690
	SVR	c = 1, PUK kernel	0.4203	0.4020	0.9672	0.9716
Jangali	Fitted model	FW = 0.0005(L×D^2^) − 0.0375	0.0822	0.1139	0.9427	0.9242
	MLR	FW = 0.101 L + 0.200D -2.791	0.0980	0.1293	0.9185	0.9046
	MLP	Topology: 2-11-1	0.0903	0.1124	0.9337	0.9278
	DT	M5P Tree	0.0848	0.1068	0.9392	0.9330
	SVR	c = 1, PUK kernel	0.0844	0.1025	0.9396	0.9394

Another important note from the evaluated models in this study is that all the optimal SVR models had a PUK kernel, while all the optimal decision tree models were of the M5P type. The PUK kernel exhibits excellent flexibility and can serve as a versatile kernel for SVMs, outperforming common kernels such as linear and RBF kernels, in both classification and regression tasks^53^. The terminal nodes of M5P trees consist of linear regression functions, allowing M5P trees to generate continuous numerical values and be applicable to regression problems^52^.

In addition to the success of the machine learning models, these results demonstrate the natural characteristics of the plum fruit, enabling the non-destructive determination of its weight based solely on its two-dimensional components.

### Validating the generality of 2019 models

**Fig. 12:**
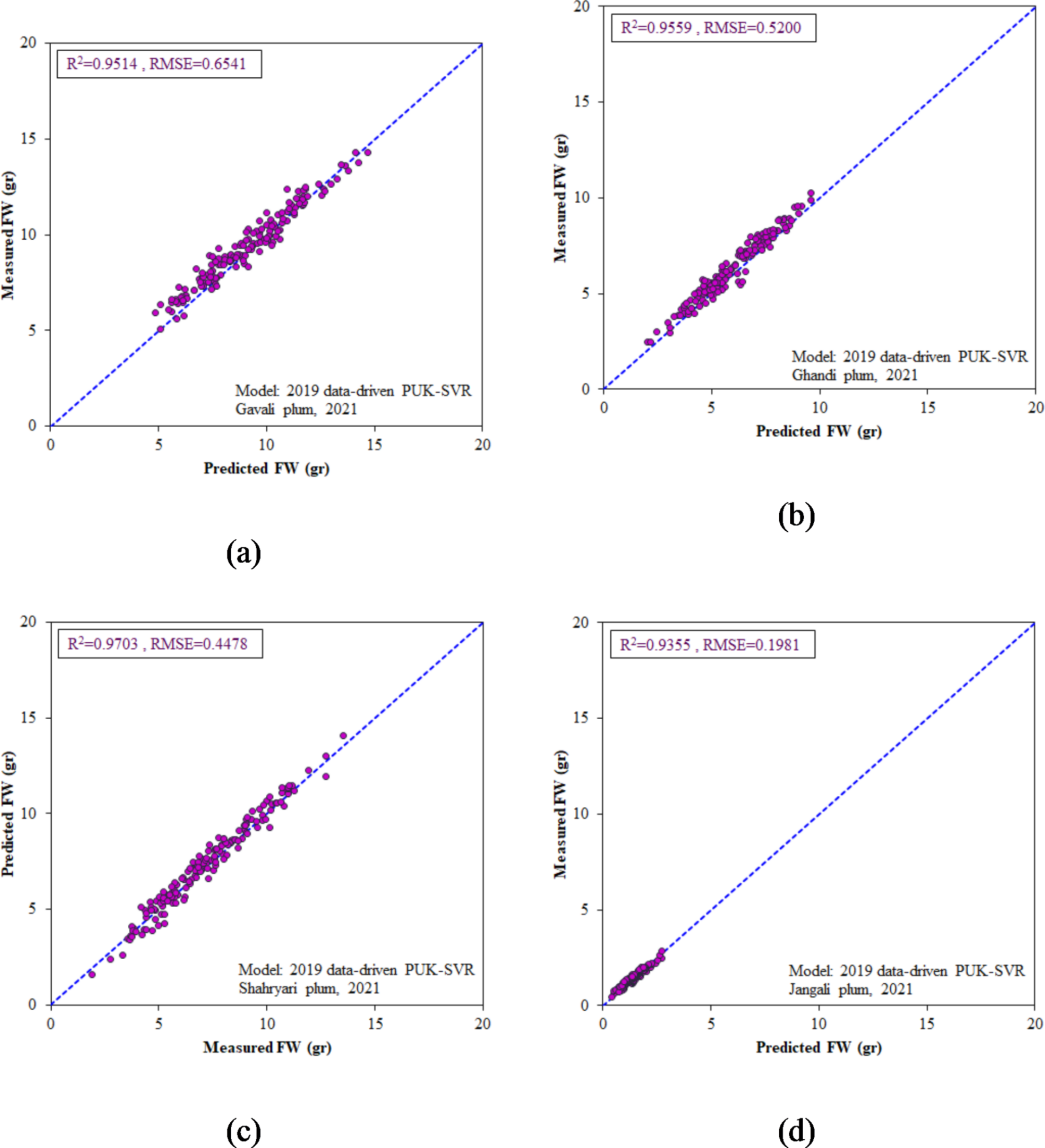
Scatter plots of 2019 optimal PUK-SVR model for estimating the FW of four plum genotypes in 2021 datasets; (**a**) Gavali genotype, (**b**) Ghandi genotype, (**c**) Shahryari genotype, and (**d**) Jangali genotype.

As stated in the materials and methods section, to validate the generalization ability and applicability of the selected 2019 models, we also conducted data in 2021. The length, diameter, and weight of the fruits were measured for the same genotype, two other genotypes from *Prunus domestica*, and another genotype, named myrobalan plum (*Prunus cerasifera*). The optimal models from 2019 were used to predict FW of these four plum genotypes. The regression parameters of R^2^, and RMSE between the modeled and measured FWs for the four genotypes are presented in Table 5. Interestingly, the selected 2019 models not only accurately predicted FW for the same plum accession but also successfully estimated FW for the other three genotypes. In this regard, the PUK-SVR model excels in accurately estimating the weight of various plum genotypes (0.9355 < R^2^ < 0.9703 and 0.1981 gr < RMSE < 0.6541 gr). The actual vs. PUK-SVR generated FW values for the four genotypes are depicted in Fig. 12. The FW data points do not significantly deviate from the line of equality, confirming the high predictive performance of the PUK-SVR model.

It should be noted that the fitted model based on the 2019 dataset, FW = 0.0225(L×D) − 4.4940, ranked second in predicting FW on the 2021 datasets, with slightly lower R^2^ values and higher RMSE values than the PUK-SVR model. In addition to its high performance (0.9301 < R^2^ < 0.9681 and 0.2138 gr < RMSE < 0.6895 gr), the FW = 0.0225(L×D) − 4.4940 model offers high applicability and accessibility to users, enabling them to calculate fruit weight based on length and diameter through a simple mathematical relation.

**Table 5:** Validation results of 2019 optimal models for estimating the FW of four plum genotypes in 2021 datasets.

Plum type:	Gavali		Ghandi		Shahryari		Jangali	
Model specification	RMSE (gr)	R^2^	RMSE (gr)	R^2^	RMSE (gr)	R^2^	RMSE (gr)	R^2^
Fitted: FW = 0.0225(L×D)-4.4940	0.6895	0.9501	0.5626	0.9446	0.4506	0.9681	0.2138	0.9301
MLR: FW = 0.479 L + 0.644D -18.346	0.8263	0.9373	0.7784	0.9206	0.6384	0.9437	0.2623	0.9165
MLP: Topology: 2-3-1	0.8661	0.9390	0.8501	0.9120	0.7702	0.9418	0.3025	0.9134
DT: M5P Tree	0.7996	0.9373	0.7782	0.9206	0.6388	0.9437	0.3217	0.9165
**SVR: c = 0.1**,** PUK kernel**	**0.6541**	**0.9514**	**0.5200**	**0.9559**	**0.4478**	**0.9703**	**0.1981**	**0.9355**

### Comparison with previous studies

The relationship between the fresh weight and diameter of blueberry fruit was found to be consistent across different genotypes and growth seasons in a study by Jorquera-Fontena, et al.^54^. They discovered an allometric relationship between berry diameter and weight. Based on the findings of our research, it is quite feasible to determine the weight of plum fruits using only their image-extracted dimensional measures in an efficient, rapid, and non-invasive manner.

Phate, et al.^55^ reported that using the SVR model, the weight of sweet lime can be estimated with high accuracy (R^2^ of 0.9867 and 0.9866 during model training and testing, respectively) based on its geometrical attributes extracted by a computer vision system. The findings of our study align with those reported by Saglam and Cetin^56^ regarding the successful use of machine learning for predicting the mass of pistachio cultivars based on their shape and size attributes. Similarly, our results are consistent with those presented by Abdel-Sattar, et al.^57^ on the development of SVR and ANN models for accurately predicting the mass of Ber fruits based on their axial dimensions.

Basak et al. (2022) utilized image processing and machine learning models to non-destructively estimate the weight of strawberries. They developed both LR and SVR models using pixel numbers from fruit images as input parameters. Their findings indicated that the LR model performed slightly better than the SVR model, with a maximum R^2^ of 96.3% during training and 89.6% during testing.

We recommend expanding the application of ML and image-based methods for fruit and vegetable weight FW estimation beyond plums. ML models, such as SVR and ANNs, combined with image processing, offer potential for non-destructive FW estimation across various horticultural crops, including apples, tomatoes, and citrus fruits. This approach improves prediction accuracy and enables real-time monitoring and quality control. Mobile-based systems integrating advanced ML algorithms and imaging could further streamline this process for farmers and agronomists. Future research should explore incorporating factors like fruit density, type, and ripeness to enhance model robustness and accuracy across diverse fruit types and growing conditions.

### Limitations of our proposed methodology

The proposed methodology for estimating fruit weight using smartphone images has some limitations. Variations in natural lighting conditions can affect image quality and consistency, introducing variability in the image processing and feature extraction stages. Background interference in natural settings can complicate the segmentation process, as a uniform and non-reflective background is crucial for accurate analysis. Maintaining consistent camera positioning, as done in the controlled laboratory environment, is challenging in the field and can lead to measurement errors. The orientation of the fruit during image capture can also impact the visibility of its dimensions, affecting accuracy. Additionally, environmental factors such as wind, rain, or dust can degrade image quality and overall feasibility. Addressing these limitations in future research could involve developing standardized protocols for field image capture, implementing advanced image processing techniques, and exploring additional sensors or imaging technologies to enhance accuracy and reliability.

## Conclusion

Monitoring FW is crucial in horticultural production systems. Indirectly determining FW without separating it from the tree branch enables continuous extraction of information about fruit and tree health over time. This study utilized image processing and ML methods to indirectly estimate the weight of plum fruits. Data were collected in 2019 for model development and in 2021 for model validation. The PUK-SVR model was found to be the best estimator for the weight of plum fruits. It achieved R^2^ values of 0.9369 and 0.9267 during the training and test stages, respectively. The model’s generality was tested on data from four different plum varieties collected in 2021, and the resulting R^2^ values were above 0.9355. Based on the results of the research, it was concluded that plum fruit weight can be accurately and quickly determined using appropriate image processing and machine learning methods. Considering the non-destructive nature of machine vision systems and the use of a mobile camera in this research, the study provides valuable information for developing a mobile-based system to non-destructively monitor plum FW. While our study successfully demonstrated the use of fruit dimensions for predicting plum weight, future research could enhance these models by incorporating additional factors such as fruit density, type, and degree of ripeness. Integrating these variables could potentially improve the accuracy and robustness of the weight predictions. Additionally, adapting this method to other fruits with varying morphologies requires consideration of factors like species-specific density variations, seasonal changes, and post-harvest conditions (e.g., moisture content and density fluctuations over time). Exploring advanced AI techniques and employing near-infrared or hyperspectral imaging could enable the model to account for these characteristics, further validating and refining the predictive models. Utilizing larger, more diverse datasets would also help make these models more universally applicable across different fruit types and growing conditions.

## Data Availability

All datasets used and/or analyzed in this study can be found in the article, Tables, and Figures shared.
